# Correlation analysis of epicardial adipose tissue thickness, C-reactive protein, interleukin-6, visfatin, juxtaposed with another zinc finger protein 1, and type 2 diabetic macroangiopathy

**DOI:** 10.1186/s12944-021-01451-7

**Published:** 2021-03-15

**Authors:** Yuan-Yuan Gong, Hai-Ying Peng

**Affiliations:** 1 Department of Endocrinology, Qingdao West Coast New Area Central Hospital, Qingdao, China; 2 Department of Special Examination, Shandong Provincial Third Hospital, Shandong University, No 11 Wuying Mountain Middle Road, Tianqiao Distrct, Jinan, 250031 China

**Keywords:** Epicardial adipose tissue thickness, C-reactive protein, Interleukin-6, Visfatin, Juxtaposed with another zinc finger protein 1, Type 2 diabetic macroangiopathy, Correlation

## Abstract

**Background:**

To investigate the correlation between the thickness of epicardial adipose tissue (EAT), C-reactive protein (CRP), interleukin (IL) -6, visfatin, juxtaposed with another zinc finger protein 1 (JAZF1) and type 2 diabetic mellitus (T2DM) macroangiopathy.

**Methods:**

The study enrolled 82 patients with T2DM with macroangiopathy (the Complication Group), and 85 patients with T2DM (the Diabetes Group) who were admitted to Shandong Provincial Third Hospital from February 2018 to February 2020. In addition, 90 healthy people who underwent physical examination at the same hospital during the same period were enrolled (the Healthy Control Group). Age, gender, height, weight, waist circumference (WC), hip circumference (HC), diabetic course and therapeutic drugs, waist hip ratio (WHR), and body mass index (BMI) were recorded and calculated.

**Results:**

The baseline characteristics of the three groups were comparable, and the diabetic course of the Complication Group and the Diabetes Group was not significantly different (*P* > 0.05). The WHR of the Complication Group was higher than that of the Diabetes Group and the Healthy Control Group, with statistical significance (*P* < 0.05). The FPG, 2hPG, HbA1C, CRP, IL-6, Visfatin, JAZF1, HOMA-IR, EAT thickness, and baPWV of the Complication Group were all higher than those of the Diabetes Group and the Healthy Control Group (*P* < 0.05, respectively). The JAZF1 and FIns of the Complication Group and Diabetes Group were lower than those of the Healthy Control Group, and JAZF1 of the Complication Group was lower than the Diabetes Group with statistical significance (*P*<0.05, respectively). Pearson correlation analysis showed that the EAT thickness was positively correlated with CRP, IL-6, visfatin, and JAZF1 (*r* = 0.387, 0.451, 0.283, 0.301, respectively, all *P*<0.001). Pearson correlation analysis showed that baPWV was positively correlated with EAT thickness, CRP, IL-6, visfatin, and JAZF1 (*r* = 0.293, 0.382, 0.473, 0.286, respectively, all *P* < 0.001). Multivariate stepwise regression analysis showed that FPG, 2hPG, HbA1C, CRP, IL-6, visfatin, JAZF1, and EAT thickness were independent risk factors that affected T2DM macroangiopathy.

**Conclusions:**

Clinical monitoring and treatment of T2DM macroangiopathy can use CRP, IL-6, Visfatin, JAZF1, and EAT thickness as new targets to delay the progression of the disease. Further research on the relationship between the above factors and the pathogenesis of T2DM macroangiopathy may be helpful provide new treatment strategies.

## Introduction

Type 2 diabetes mellitus (T2DM) is a common metabolic disease in endocrinology department. Its main feature is chronic hyperglycemia, accompanied by insulin resistance and islet β-cell damage. With continuous improvement of the socio-economic level, the prevalence of T2DM has been increasing globally in recent years and is showing a younger trend [1]. An epidemiological study showed that the number of patients with T2DM in the world reached 285 million in 2009, and is estimated to reach 552 million in 2020 [2]. On this trend, the number of T2M patients may rise to 629 million by 2045 [3]. A study showed that patients with T2DM were often accompanied by complications such as macroangiopathy, eye disease, and renal failure, the most common of which was macroangiopathy, which accounted for about 75% of T2DM complications [4] Macroangiopathy is not only the main cause of disability in patients, but also can lead to death of patients, posing a serious threat to the life and health of patients. Therefore, how to effectively prevent and treat type 2 diabetic macroangiopathy is one of the key clinical issues that need to be solved urgently.

Macroangiopathy includes coronary heart disease, hypertension, cerebrovascular disease, and vascular disease of lower extremity. The main pathological basis is atherosclerosis. The thickness of epicardial adipose tissue (EAT) is closely related to coronary atherosclerosis and can reflect plaque severity [5]. A recent study has confirmed that inflammation may play an important role in the pathogenesis of type 2 diabetic macroangiopathy. A variety of inflammatory factors, including C-reactive protein (CRP) and interleukin (IL) -6, not only regulate themselves and other tissues, but also are associated with insulin resistance and islet cell dysfunction, promoting the occurrence of diabetes [6]. CRP and IL-6 are important markers of local vascular injury, which can reflect the severity of inflammation. The EAT is located between the visceral pericardium and the myocardium. The thickness of EAT is closely related to coronary atherosclerosis and can reflect the severity of atherosclerosis [6]. Juxtaposed with another zinc finger protein 1 (JAZF1), also known as TAK1-Interacting Protein 27, is closely related to various diseases such as atherosclerosis and T2DM [7]. Visfatin, a cytokine found in visceral fat, binds and activates insulin receptors and produces insulin-like effects. Ming et al. [8] indicated that JAZF1 promoted the expressions of visfatin, peroxisome proliferators-activated receptor (PPAR) α, and PPARβ/δ in adipocytes but simultaneously inhibited the expressions of TAK1 and PPARγ. There are few studies on the relationship between EAT thickness, CRP, IL-6, visfatin, JAZF1 and type 2 diabetic macroangiopathy. Therefore, this study aimed to explore the relationship between these factors and type 2 diabetic macroangiopathy, in order to provide a theoretical basis for the clinical treatment of type 2 diabetic macroangiopathy.

## Subjects and methods

### Clinical data

**Fig. 1:**
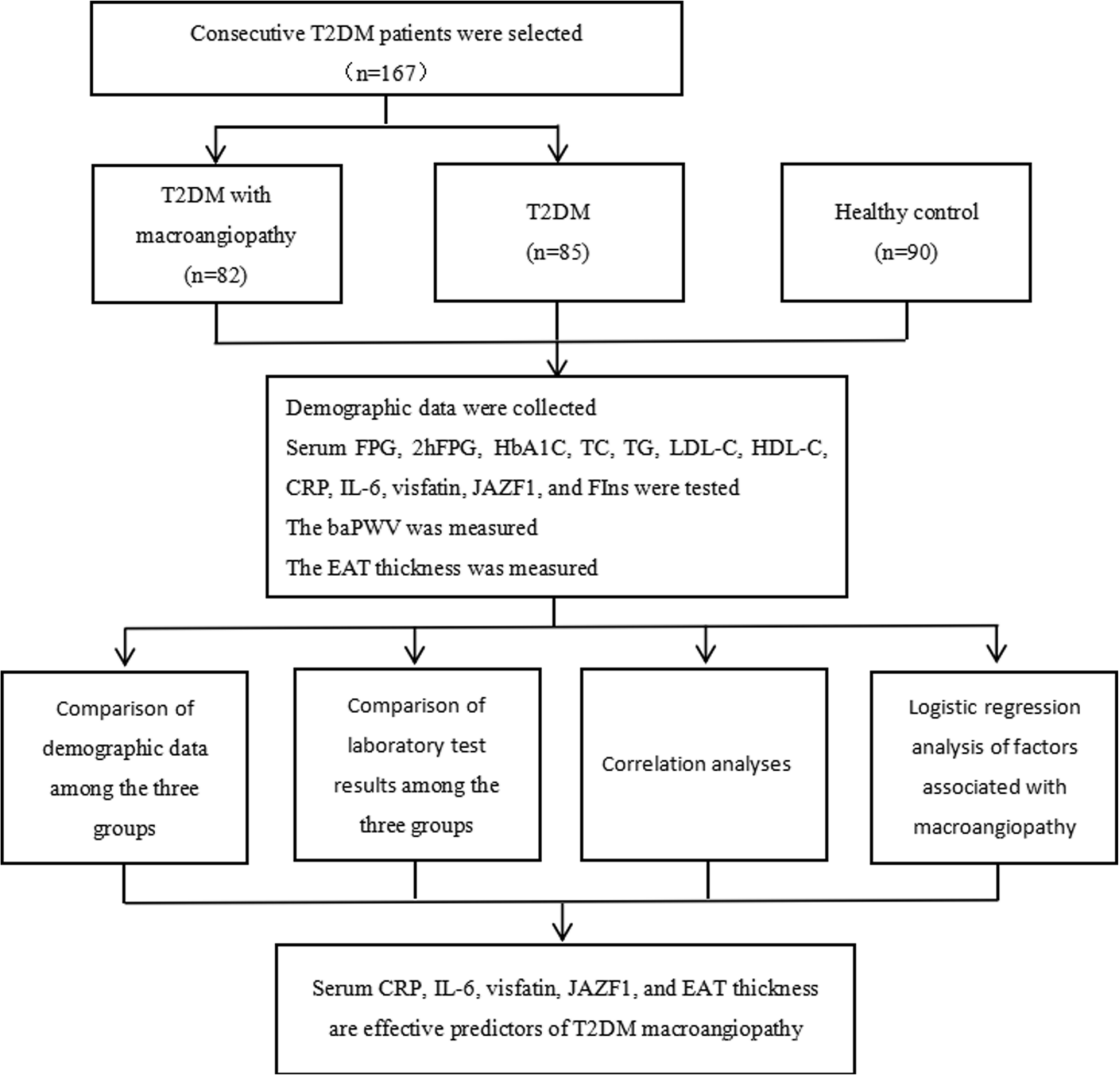
Flow chart of this study

A total of 167 T2DM patients who were hospitalized from February 2018 to February 2020 were consecutively enrolled in the study. Among them, 82 patients with diabetes with macroangiopathy were assigned to the Complication Group, and 85 patients with simple T2DM were assigned to the Diabetes Group. In addition, 90 healthy people who underwent physical examination during the same period were enrolled as the Healthy Control Group. The flow chart is shown in Fig. 1.

The study protocol was approved by the Ethics Committee of Shandong Provincial Third Hospital. The formulation of this research protocol complies with the relevant requirements of the Declaration of Helsinki of the World Medical Association.

### Inclusion and exclusion criteria

Inclusion criteria: (1) All patients with T2DM met the 2019 World Health Organization (WHO) criteria for diabetes diagnosis and classification [9]. (2) The diagnostic criteria of diabetic macroangiopathy were brachial - ankle artery pulse wave velocity (baPWV) > 1400 cm/s on either side or both sides of the lower limbs in T2DM patients [10, 11], and the patients met one of the following criteria: ① magnetic resonance imaging (MRI) or computed tomography (CT) scan of the brain revealed ischemic lesions, confirming cerebral infarction; ② coronary CT or coronary angiography confirmed coronary heart disease (more than 70% lumen stenosis in a major epicardial vessel or more than 50% in the left main coronary artery); ③ a history of coronary heart disease, old cerebral infarction or other cerebrovascular diseases; ④ Doppler ultrasonography revealed extensive irregular stenosis or segmental occlusion of lower extremity arteries; (3) those with normal functions of major organs such as liver and kidney in biochemical tests; (4) the clinical data were complete; (5) the patient and his/her relatives agreed and signed informed consent.

Exclusion criteria: (1) Patients with type 1 or other types of diabetes; (2) malignant tumor; (3) gastrointestinal lesions; (4) acute or chronic infection; (5) acute complications of diabetes; (6) hypertension (systolic blood pressure ≥ 140 mmHg, and/or diastolic blood pressure ≥ 90 mmHg); (7) a history of lower limb gangrene.

## Methods

Age, gender, height, weight, waist circumference (WC), leg circumference (HC), and diabetic course in the 3 groups were recorded. WHR and BMI were calculated.

Venous blood was collected via the elbow vein from 2 groups of diabetic patients who fasted for more than 12 h and 2-h postprandial blood was also collected. The fasting plasma glucose (FPG) and 2-h postprandial plasma glucose (2hPG) were detected by glucokinase method. Hemoglobin A_1_C (HbA_1_C) was detected by high pressure liquid chromatography [12]. Total cholesterol (TC), triglyceride (TG), high density lipoprotein cholesterol (HDL-C), low density lipoprotein cholesterol (LDL-C) and CRP were detected by Hitachi 7080 fully automated analyzer (Hitachi, Tokyo, Japan). IL-6, visfatin and JAZF1 were detected by TSZ ELISA kit (Biotang Inc./TSZ ELISA, Waltham, MA, USA). Electrochemical luminescence method was used to detect FInS. Homeostasis model assessment of insulin resistance (HOMA-IR) = FPG × FInS/22.5. The baPWV was measured using Colin VP-1000 fully automatic arteriosclerosis detector (Colin Medical Technology Co., Komaki, Japan) and the OMRON HEM-9000AI device (Omron Healthcare, Kyoto, Japan). To ensure accuracy, the mean value was taken of 5 consecutive measurements.

**Fig. 2:**
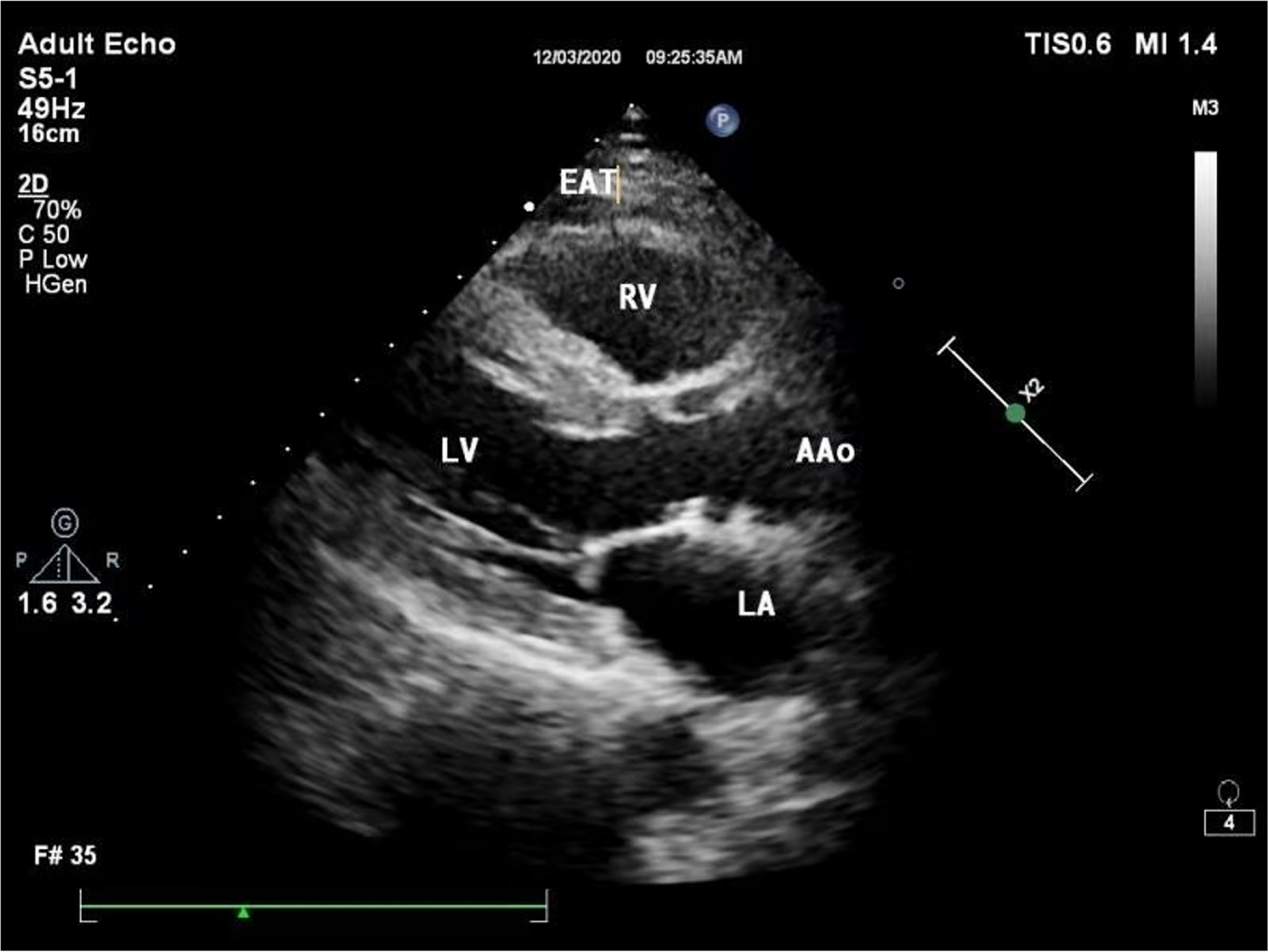
The thickness of EAT was measured by ultrasound. In the parasternal long-axis view, the EAT thickness of the free wall of the right ventricle was measured at the end of diastole. EAT: epicardial adipose tissue; RV: right ventricular; LV: left ventricular; AAo: ascending aorta; LA: left atrium

Measurement of EAT thickness [13]: Vivid 7 (GE Healthcare, Milwaukee, WI, USA) and EPIQ5 (Philips, Amsterdam, the Netherlands) Echocardiography Machine was used with a 2-4 MHz cardiac probe. The cardiac probe was connected to the electrocardiogram (ECG), and the end of P wave was judged as the end of diastole. The patients were asked to lie on the left side. The thickest at the anterior wall of the right ventricle along the long axis of the parasternal left ventricle was measured at the end of the diastole (Fig. 2). The above operations were performed by physicians who had worked in the imaging department for more than 8 years. To ensure accuracy, the operations were performed consecutively for 5 times and the average value was taken.

### Statistical analysis

Data were statistically analyzed using statistical software SPSS 23.0. Normally distributed data were represented by (mean ± standard deviation), and analyzed using the t-test. Non-normally distributed measurement data were represented by median and interquartile range [M (Q_L_, Q_U_)], and analyzed using Wilcoxon rank-sum test. One-way ANOVA was used for comparison among multiple groups using post-hoc test. Categorical data were expressed as counts and percentages and analyzed using χ^2^ test. Pearson analysis was used for correlation analysis. Spearman test was used to analyze the correlation of non-normally distributed data. The determinants of T2DM macroangiopathy were analyzed by logistic regression. The level of statistical significance for all the above tests was defined at a probability value of less than 0.05 (*P* < 0.05).

## Results

### Demographic characteristics

The demographic data of the three groups were as follows: The Complication Group: The gender distribution was 39 males, and 43 females. The age range was 47–75 years, with an average age of 55.4 ± 5.2 years. The BMI was 25.02 ± 2.28 kg/m^2^. The Diabetes Group: The gender distribution was 45 males and 40 females. The age range was 48–76 years, with an average age of 55.9 ± 5.3 years. The BMI was 24.79 ± 2.12 kg/m^2^. The Healthy Control Group: The gender distribution was 47 males, and 43 females. The age range was 47–78 years, with an average age of 56.1 ± 6.2 years. The BMI was 24.02 ± 2.39 kg/m^2^. The baseline characteristics were comparable among the three groups (*P* > 0.05). The WHR of the Complication Group was higher than that of the Diabetes Group and the Healthy Control Group, with statistical significance (*P* < 0.05) (Table 1).

**Table 1:** Demographic characteristics

	Complications Group (*n* = 82)	Diabetes Group (*n* = 85)	Healthy Control Group(*n* = 90)	χ^2^/t	*P* value
Gender (male/female)	43/39	45/40	47/43	0.009	0.996
Age (years)	55.4 ± 5.2	55.9 ± 6.2	56.1 ± 6.2	0.355	0.702
WHR	1.03 ± 0.03^a,b^	0.95 ± 0.05	0.91 ± 0.06	133.612	<0.001
BMI (kg/m^2^)	25.02 ± 2.28	24.79 ± 2.12	24.02 ± 2.39	0.331	0.719
Duration of diabetes (years)	4.03 ± 0.38	3.97 ± 0.41	–	0.981	0.328
Medications for diabetes and dyslipidemia				1.428	0.629
Insulin	33 (37.80)	33 (38.82)			
Dimethyldiguanide	29 (35.37)	27 (31.76)			
Alpha-glucosidase inhibitor	15 (18.29)	28 (32.94)			
Statins	20 (24.39)	28 (32.94)			

^a^compare with Diabetes Group (*P*<0.05); ^b^compare with Healthy Control Group (*P*<0.05); *WHR* waist hip ratio, *BMI* body mass index

### Comparison of laboratory test results among the three groups

There was no statistical difference in TC, HDL-C, and LDL-C among the 3 groups (*P* > 0.05). The FPG, 2hPG, HbA_1_C, CRP, IL-6, visfatin, JAZF1, HOMA-IR, baPWV, and EAT thickness of the Complication Group were all higher than those of the Diabetes Group and the Healthy Control Group, and the JAZF1 and FIns of the Complication Group and the Diabetes Group were lower than those of the Healthy Control Group, with statistical significance (*P* < 0.05, respectively), JAZF1 of the Complication Group was lower than that of the Diabetes Group (*P* < 0.05). The FPG, 2hPG, TG, HbA_1_C, CRP, IL-6, visfatin, JAZF1, HOMA-IR, baPWV, and EAT thickness of the Diabetes Group were all higher than those of the Healthy Control Group, with statistical significance (*P* < 0.05). There was no statistical difference in TG and FIns between the Complication Group and the Diabetes Group (*P*>0.05) (Table 2).

**Table 2:** Comparison of laboratory test results among three groups

	Complication Group (*n* = 82)	Diabetes Group (*n* = 85)	Healthy Control Group (*n* = 90)	χ^2^/t	*P*
FPG (mmol/L)	12.45 ± 1.29^a,b^	9.39 ± 1.04^b^	5.39 ± 0.87	936.87	<0.001
HbA_1_C (%)	9.81 ± 0.17^b^	9.01 ± 0.26^b^	5.56 ± 0.54	3328.435	<0.001
TC (mmol/L)	4.78 ± 1.17	4.69 ± 1.12	4.39 ± 1.02	2.988	0.052
TG (mmol/L)	2.38 ± 0.13^b^	2.33 ± 2.08^b^	1.79 ± 0.37	6.315	0.002
HDL-C (mmol/L)	1.17 ± 0.41	1.11 ± 0.37	1.03 ± 0.37	2.897	0.057
LDL-C (mmol/L)	3.02 ± 0.98	2.85 ± 0.91	2.76 ± 0.91	1.704	0.184
CRP (mg/mL)	6.78 ± 0.47^a,b^	3.45 ± 0.21^b^	2.49 ± 0.24	4071.146	<0.001
IL-6 (pg/mL)	123.93 ± 30.93^a,b^	80.99 ± 12.39^b^	64.39 ± 8.73	209.132	<0.001
Visfatin (pg/mL) [M (Q_L_,Q_U_)]	667.93 (340.83,1093.89)^a,b^	372.79 (334.93,470.93)^*^	358.73 (332.98,428.71)	183.09	<0.001
JAZFl (pg/mL) [M (Q_L_,Q_U_)]	122.29 (86.39,253.29)^a,b^	153.39 (132.91,203.29)^b^	221.92 (204.92,341.92)	208.11	<0.001
FIns (μIU/mL)	12.09 ± 1.21^b^	12.06 ± 1.34^b^	15.41 ± 2.01	131.363	<0.001
HOMA-IR	6.54 ± 0.37^a,b^	4.62 ± 0.36^b^	3.56 ± 0.32	1586.769	<0.001
EAT thickness (mm)	5.58 ± 1.23^a,b^	3.58 ± 0.78^b^	2.71 ± 0.54	233.854	<0.001
baPWV	1698.23 ± 227.89^a,b^	1607.46 ± 236.41^a,b^	1320.28 ± 112.13	96.625	< 0.001

*FPG* fasting plasma glucose, *HbA*_*1*_*C* hemoglobin A_1_C, *TC* total cholesterol, *TG* triglyceride, *HDL-C* high density lipoprotein cholesterol, *LDL-C* low density lipoprotein cholesterol, *CRP* C-reactive protein, *IL-6* interleukin-6, *FIns* fasting insulin, *HOMA-IR* homeostasis model assessment of insulin resistance, *EAT* epicardial adipose tissue

^a^compare with Diabetes Group (*P*<0.05); ^b^compare with Healthy Control Group (*P*<0.05)

### The correlation between EAT thickness and CRP, IL-6, visfatin, and JAZF1 in the complication group

Correlation analysis revealed that EAT thickness was positively correlated with CRP, IL-6, visfatin, and JAZF1 (*r* = 0.387, 0.451, 0.283, 0.301, respectively, *P* < 0.001).

### The correlation analysis between baPWV and EAT thickness, CRP, IL-6, visfatin, and JAZF1 in the complication group

Correlation analysis revealed that baPWV was positively correlated with EAT thickness, CRP, IL-6, visfatin and JAZF1 (*r* = 0.293, 0.382, 0.473, 0.286, respectively, *P*<0.001).

### Logistic regression analysis of factors associated with macroangiopathy in T2DM

As can be seen from Table 3, EAT was correlated with multiple indicators. In order to control the influence of confounding factors and possible collinearity between independent variables, multiple logistic regression analysis was performed of risk factors of T2DM macroangiopathy patients. BaPWV> 1400 cm/s [14] was assigned as the dependent variable, and FPG, 2hPG, HbA1C, TC, TG, HDL-C, LDL-C, CRP, IL-6, visfatin, JAZF1, FIns, HOMA-IR, and EAT thickness were assigned as the independent variables. The results showed that FPG, 2hPG, HbA1C, CRP, IL-6, visfatin, JAZF1, and EAT thickness were all factors that associated with T2DM macroangiopathy. The R2 value of this regression model was 0.892.

**Table 3:** Logistic regression analysis of influencing factors of T2DM macroangiopathy

	Regression coefficient	Wald χ^2^	OR	95%*CI*	*P* value
Age	1.787	20.252	1.165	1.016–1.411	0.024
Gender	0.127	4.641	1.183	1.011–1.377	0.002
BMI	0.899	11.372	2.447	1.456–4.231	0.001
FPG	3.387	8.219	27.932	2.779–268.93	0.003
2hPG	3.287	8.321	28.021	2.701–254.31	0.004
HbA_1_C	3.192	8.021	26.932	2.672–251.01	0.001
TC	1.012	2.193	6.382	0.998–41.921	0.056
TG	1.009	2.008	6.482	0.893–37.632	0.059
HDL-C	1.129	2.231	6.504	0.894–39.841	0.063
LDL-C	1.273	2.573	6.631	0.763–38.913	0.067
CRP	2.763	5.743	15.032	1.559–138.931	0.009
IL-6	2.893	5.632	14.392	1.372–136.49	0.008
Visfatin	3.832	8.932	29.031	2.713–297.32	0.002
JAZF1	2.381	5.193	14.873	1.382–133.02	0.012
FIns	1.231	2.237	6.449	0.872–38.932	0.064
HOMA-IR	3.028	2.087	6.218	0.832–38.932	0.053
EAT thickness	2.932	5.271	15.932	1.453–139.872	0.006

*OR* odds ratio, *CI* confidence interval, *BMI* body mass index, *FPG* fasting plasma glucose, *HbA*_*1*_*C* hemoglobin A_1_C, *TC* total cholesterol, *TG* triglyceride, *HDL-C* high density lipoprotein cholesterol, *LDL-C* low density lipoprotein cholesterol, *CRP* C-reactive protein, *IL-6* interleukin-6, *FIns* fasting insulin, *HOMA-IR* homeostasis model assessment of insulin resistance, *EAT* epicardial adipose tissue

## Discussion

T2DM has become the third chronic disease affecting human life after cardiovascular diseases and tumors. Macroangiopathy is one of the main complications of T2DM. It can involve medium sized or large blood vessels, leading to stenosis and occlusion of the lumen. In severe cases, it can cause plaque rupture and shedding, and induce embolism or bleeding. The mechanism may be related to factors such as glucose and lipid metabolism disorder, blood hypercoagulability, microcirculation disorder, and decline of vascular endothelial function. The pathological basis of T2DM macroangiopathy is atherosclerosis, and its mechanism may be related to factors such as glucose and lipid metabolism disorder, blood hypercoagulability, microcirculation disorder, and vascular endothelial function decline [15]. Umemura et al. [16] revealed that the prevalence of macroangiopathy in Caucasian T2DM patients is twice that of microvascular disease, and the mortality rate is 76 times that of microvascular disease. In clinical practice, controlling blood glucose alone cannot reduce the risk of macroangiopathy. Therefore, prevention of macroangiopathy in T2DM is a difficult disease of global concern and one of the important issues to be solved urgently.

The EAT is located on the surface of the myocardium. It is a special visceral fat between the epicardium and the visceral pericardium. It is an important endocrine organ of the body and a storage warehouse of body fat energy. It is mainly distributed in the free wall of the right ventricle, the apex of the left ventricle and the free wall of the right ventricle. It can release a variety of biologically active molecules at a high rate, and communicate through signal transduction between the heart, liver, vascular endothelial cells, adipose tissue, skeletal muscle and pancreatic islet cells, forming a complex regulatory network [17]. Chen et al. [18] indicated that the EAT thickness of T2DM patients is significantly higher than that of normal subjects.

CRP is the most significant clinical marker of inflammation. It has the function of recognizing and regulating immunity. It can also enhance the reactivity of leukocytes, play a firm role in the fixation of complement, and strengthen the ability to remove cell debris in inflammation sites. By activating complement, inflammatory mediators such as histamine are released [19]. Shen et al. [20] suggested that CRP is present in atherosclerosis and produces proinflammatory and atherogenic pathways, suggesting that CRP can be used not only as an inflammatory marker, but also as an independent risk factor in the pathologic formation of atherosclerosis.

IL-6 is synthesized by fibroblasts, vascular endothelial cells, activated monocytes and other cells. It can affect inflammation and host defense through cellular and humoral immune functions and is the main circulating substance in vivo that links systemic immune response with local vascular injury [21]. Ziegler et al. [22] suggested that T2DM may be a cytokine-mediated inflammatory disease, and T2DM and atherosclerosis are both inflammatory diseases. Inflammation plays an important role in the occurrence and development of chronic vascular complications and atherosclerosis and has been considered as one of the important factors in the occurrence and development of atherosclerosis. Since IL-6 can stimulate liver cells to synthesize CPR, the changes in the levels of the two in patients also have a certain correlation. Deng et al. [23] proved that hs-CRP and IL-6 have diagnostic significance for patients with T2DM vascular disease.

Li et al. [24] confirmed that the human JAZF1 gene sequence has extremely high homology with mouse gene sequence. JAZF1 is located in the nucleus and its mRNA is common in human tissues. TAKI is an orphan nuclear receptor that plays a role in multiple metabolism-related genes, and has a regulatory effect on liver lipid metabolism. Lack of TAKI in mice can reduce the inflammation of adipose tissue, loss of mitochondrial function, reduce the formation of CD36 and foam cells, and then cause atherosclerosis [25]. Marselli et al. [26] found that PPAR_α_-mediated transcriptional activation is inhibited by TAKI of liver cells, and PPAR_α_ regulates gene expression of multiple links in the liver, which indirectly suggests that JAZF1 can improve lipid metabolism. Animal experiment by Zhou et al. [27] indicated that JAZF1 gene overexpression can improve lipid metabolism and inhibit the accumulation of macrophages in plates, thus reducing or delaying the formation of atherosclerosis. Therefore, it is speculated that JAZF1 may play an important role in diabetic macroangiopathy, hyperlipidemia and glycolipid metabolism.

Visfatin is a factor that exists in visceral fat cells, which can combine activated insulin receptors with Insulin-Like Growth Factor, and is closely related to vascular smooth muscle maturation, atherosclerosis, immune regulation and inflammatory reactions. Ran et al. [28] have shown that inhibition of JAZF1 reduces the expression level of visfatin. However, there are few clinical reports on the correlation between EAT thickness, CRP, IL-6, Vivfatin, JAZF1 and T2DM macroangiopathy.

The results of this study showed no statistical difference in baseline characteristics among the three groups, and the diabetic course was comparable between the Complication group and the Diabetes group (*P* > 0.05). The WHR, FPG, 2hPG, HbA_1_C, CRP, IL-6, visfatin, JAZF1, HOMA-IR, and EAT thickness were all higher in the Complication Group than the Diabetes Group and the Healthy Control Group (*P* < 0.05, respectively), and the FIns of both the Complication Group and the Diabetes Group were lower than that of Healthy Control Group (*P*<0.05). It was suggested that the above indicators could predict T2DM with macrovascular lesions, especially CRP, IL-6, visfatin, JAZF1, HOMA-IR, and EAT thickness. According to the changes of the above indicators, early intervention in patients with T2DM can prevent the occurrence of disability and death to a certain extent and has important clinical significance for the treatment of T2DM macroangiopathy.

CT and MRI are the main methods to measure EAT thickness. However, due to the high price of CT and MRI, the radiation of CT and the noise of MRI, the large-scale use of CT and MRI is affected to some extent. Uygur et al. [29] have confirmed that the measurement of EAT thickness of the anterior wall of the right ventricle by chest ultrasound is consistent with the measurement results of CT and MRI. Therefore, in this study, chest ultrasound was used to measure the EAT thickness of the anterior wall of the right ventricle in the enrolled cases and healthy controls. The measurement site was the hypoechoic and anechoic region between the epicardium of the right ventricular wall and the visceral pericardium. Because of the difference in shape, the thickest part of the anterior wall of the right ventricle was measured. The results demonstrated that repeated measurements of EAT thickness at the end of the diastole showed stable results, and the measuring method was simple and reliable.

Ultrasound measurement of EAT thickness has the following advantages in predicting T2DM macroangiopathy. First, compared with the conventional method of evaluating macroangiopathy, ultrasound can measure the thickness of EAT and examine the structure and function of the heart at the same time, combining the examination to evaluate cardiac and macroangiopathy. Second, compared with CT or MRI, ultrasound examination is cheaper and easier to repeat.

Diabetes can easily lead to atherosclerosis. As atherosclerosis progresses, plaques can block the lumen and cause cardiovascular and cerebrovascular diseases. PWV is the rate of pulse conduction from the proximal end to the distal end of the arterial wall due to the expansion and retraction of the arterial wall, which can reflect the elasticity of the artery. The higher the PWV is, the harder the blood vessel wall is [30–32]. Studies have confirmed that PWV is an independent predictor of cardiovascular events [33, 34], and also pointed out that EAT is an independent risk factor for cardiovascular disease, which can affect the process of atherosclerosis through regulating inflammation. As the lesion area of coronary heart disease expands, the thickness of epicardial tissue also increases. As the human body ages, the expansion of elastic arteries decreases while the compliance of muscular arteries increases. The baPWV measurement includes elastic arteries and muscular arteries, which more comprehensively reflects the condition of arteriosclerosis. Pearson correlation analysis found that EAT thickness was positively correlated with CRP, IL-6, visfatin, and JAZF1 (*P* < 0.001), and baPWV was positively correlated with EAT thickness, CRP, IL-6, visfatin, and JAZF1 (*P* < 0.001), suggesting that in T2DM macroangiopathy patients, EAT thickness is closely related to inflammation and lipid metabolism. With increase in EAT thickness, the levels of CRP, IL-6, visfatin, and JAZF1 also increase, which can induce hyperglycemia, insulin resistance, and vascular endothelial dysfunction, etc., promoting the occurrence and development of atherosclerosis, leading to macroangiopathy.

A previous study found a stronger link between pericardial adipose tissue and visceral abdominal adipose tissue than other cardiovascular risk factors. Vascular calcification was associated with intrathoracic and pericardial adipose tissue, probably due to a local toxic effect on the vasculature [35]. In addition, excessive serum free fatty acids (FFA) can increase glycogen and basal insulin secretion and reduce liver insulin inactivation, resulting in hyperglycemia and insulin resistance. Diabetic hyper-FFAemia can cause vascular endothelial dysfunction through inflammation, oxidative stress pathways, and mitochondrial dysfunction, and vascular endothelial dysfunction is the initiating factor leading to atherosclerosis [36]. In the pathophysiology of atherosclerosis, inflammatory mechanisms play an important role. Persistent chronic inflammatory responses lead to damage to blood vessels, causing atherosclerosis, and plaque rupture and thrombosis [37]. He et al. [38] indicated that inflammatory factors such as TNF-α and CRP are involved in the pathophysiological process of vascular disease in patients with T2DM in plateau areas. Zhuo et al. [39] found that serum JAZF1 combined with fasting C-peptide has certain value in the diagnosis of T2DM macroangiopathy. Another study has also found that increased levels of visfatin are closely related to the severity of atherosclerotic peripheral arterial obstructive disease [40]. Chang et al. [41] confirmed that visfatin is elevated in the serum of Uyghur diabetic patients.

The results indicate that FPG, 2hPG, HbA1C, CRP, IL-6, Visfatin, JAZF1, FIns, HOMA-IR and EAT thickness are all factors affecting T2DM macroangiopathy. Therefore, CRP, IL-6, visfatin, JAZF1, and EAT thickness can be used as new targets for monitoring and treating macrovascular changes in T2MD.

### Study strength and limitations

This study prospectively observed the relationship between the indicators like the EAT thickness and the incidence of T2DM macroangiopathy, and found some valuable positive indicators, which had a certain predictive value for the incidence of T2DM macroangiopathy. This study also has certain limitations. Firstly, this is a single-center trial, and selection bias cannot be completely eliminated. In addition, due to the limited time of this study, it is still unclear whether there is a cross regulation between serum CRP, IL-6, Visfatin, JAZF1, and EAT thickness, and the specific regulation mechanism still remains unelucidated which needs to be confirmed by further study. Thirdly, there are the inter- or intra- operator differences in the measurement, which may lead to a bias of EAT thickness results.

## Conclusion

CRP, IL-6, Visfatin, JAZF1, and EAT thickness are closely related to the clinical progression of patients with T2DM, which are independent risk factors for T2DM macroangiopathy. However, how these above factors affect T2DM macroangiopathy and the underlying mechanism remain to be further explored. Therefore, the above indicators, as new targets for delaying disease progression, can provide more valuable references for clinical treatment strategies.

## Data Availability

The datasets generated and analyzed during the current study are available from the corresponding author on reasonable request.

## Abbreviations

EAT: Epicardial adipose tissue
CRP: C-reactive protein
(IL)-6: Interleukin
JAZF1: Juxtaposed with another zinc finger protein 1
T2DM: Type 2 diabetic mellitus
WC: Waist circumference
HC: Leg circumference
WHR: Waist hip ratio
BMI: Body mass index
ECG: Electrocardiogram
FPG: Fasting plasma glucose
2hPG: 2 h postprandial plasma glucose
HbA1C: Hemoglobin A_1_C
TC: Total cholesterol
TG: Triglyceride
HDL-C: High density lipoprotein cholesterol
LDL-C: Low density lipoprotein cholesterol
FIns: fasting insulin
baPWV: brachial-ankle pulse wave velocity
PPAR: Peroxisome proliferators-activated receptor
MRI: Magnetic resonance imaging
